# Cancer of the supernumerary ovary in Mayer-Rokitansty-Küster-Hauser Syndrome: A case report

**DOI:** 10.3892/ol.2012.1073

**Published:** 2012-12-12

**Authors:** HYO SOOK BAE, MIN JI RYU, IN SUN KIM, SUN HAENG KIM, JAE YUN SONG

**Affiliations:** 1 Departments of Obstetrics and Gynecology, Korea University Anam Hospital, Korea University College of Medicine, Sungbuk-gu, Seoul 136705, Republic of Korea; 2 Pathology, Korea University Anam Hospital, Korea University College of Medicine, Sungbuk-gu, Seoul 136705, Republic of Korea

**Keywords:** Mayer-Rokitansty-Küster-Hauser syndrome, supernumerary ovary, ovarian cancer, pelvic mass, amenorrhea

## Abstract

Mayer-Rokitansty-Küster-Hauser (MRKH) syndrome is a Müllerian anomaly that presents with varying degrees of uterovaginal aplasia and is secondarily associated with cervicothoracic, auditory and skeletal anomalies. However, MRKH syndrome patients have normal and functional ovaries. A supernumerary ovary is an extremely rare form of an ectopic ovary and there are no reported cases of MRKH syndrome with cancer of the supernumerary ovary in the current literature. A 31-year-old female with a history of MRKH syndrome that was diagnosed 4 years previously presented with abdominal pain and a suspected malignant pelvic mass was identified. During the staging surgery, both ovaries were separated from the main mass, observed and removed. A third ovary was discovered in the pelvic mass and the diagnosis of primary ovarian cancer from the third ovary was confirmed by immunohistochemistry. We report the first known case of cancer of the supernumerary ovary in a patient with MRKH syndrome. Although both ovaries were confirmed to be normal in the patient with MRKH syndrome, we propose that an ovarian neoplasm should be considered in the diagnosis of a pelvic mass.

## Introduction

Mayer-Rokitansky-Küster-Hauser (MRKH) syndrome is characterized by congenital aplasia of the uterus and the upper two thirds of the vagina in females demonstrating normal development of secondary sexual characteristics and a normal 46, XX karyotype. MRKH may be isolated but it is more frequently associated with renal, vertebral and, to a lesser extent, auditory and cardiac defects. The first indication of MRKH syndrome is a primary amenorrhea in young females that otherwise present with the normal development of secondary sexual characteristics and normal external genitalia, with normal and functional ovaries (1).

The supernumerary ovary is an ectopic ovary that is not connected to the utero-ovarian, broad or infundibulopelvic ligaments. This is one of the rarest gynecological abnormalities (2). A supernumerary ovary in MRKH syndrome is extremely rare. A search of Google Scholar, Medscape and PubMed revealed only one case with a benign tumor in the literature (3). Therefore, we describe the first case of cancer of the supernumerary ovary. Written informed patient consent was obtained from the patient.

## Case report

### Patient

A 31-year-old nullipara was admitted to the Korea University Anam Hospital, Korea University College of Medicine (Seoul, Korea) with a 2-week history of intermittent pain in the lower abdomen and back. On ultrasound examination, an 11.8×8.3-cm cauliflower-like mass was noted on the left side of the pelvic cavity. A computed tomography (CT) scan revealed an ill-defined, irregular soft tissue mass with extensive calcification in the pelvic cavity.

The patient had been admitted to our hospital 4 years previously with chief complaints of primary infertility and amenorrhea. On admission, the patient was observed to be age-appropriate with normal development of the breasts, pubic hair and external genitalia. The karyotype was normal 46, XX. A diagnostic laparoscopy revealed an absent upper vagina and two irregularly shaped uterine bodies positioned near each of the infundibulopelvic ligaments. Both ovaries were normal in appearance. A hypoplastic and non-functioning left kidney was diagnosed, as the left kidney was not visualized in the renal scan. Additionally, a scoliotic change in the thoracic spine was observed by a chest X-ray. After two months, the patient underwent an Abbé-McIndoe procedure.

At the time of the current admission, the laboratory results were within the normal limits, with the exception of an elevated CA 125 level of 1,870.0 IU/ml. During surgery, a 25-cm pelvic mass was observed to occupy the left pelvic cavity. Multiple peritoneal seeding, omental invasion and rectal wall adhesions were detected. Following adhesiolysis, the bilateral ovaries and salpinges were demonstrated to be slightly enlarged, but normal in their contour and position. The uterus had enlarged since the previous surgery. A pelvic exenteration, omentectomy, sigmoid colon resection en-bloc, bilateral pelvic lymph node dissection and right external iliac node sampling were performed with a peritoneal washing cytology.

Both ovaries exhibited normal histological features, but with numerous carcinoma cell implants on their surface. Furthermore, the pelvic mass itself contained areas of well-defined ovarian tissue with cystic follicles. This latter finding and the immunohistochemistry results revealed that a serous tumor had developed from a third ovary, as opposed to the mesothelium.

The patient received post-operative chemotherapy. Sixteen months post-surgery, the patient was stable and did not demonstrate signs of recurrence.

### Pathological findings

**Figure 1.**
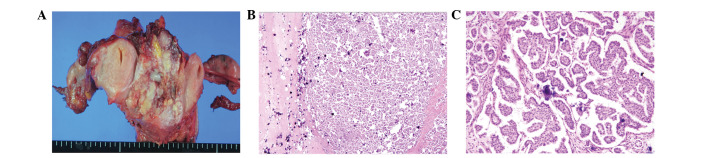
(A) The cut surface of the resected pelvic mass and organ shows double uterine corpus, and bilateral ovaries and salpinges (B and C) Multiple tumor nodules are located on the ovarian surface. The tumor is composed of numerous papillary growing tumors with psammoma bodies (B, ×40 and C, ×100).

The en-bloc resected pelvic mass consisted of an ill-defined tumor mass, a segmentallyresected rectum and duplicate uterine bodies with attached bilateral adnexae (Fig. 1). On dissection, the right uterus measured 4.7×2.0×1.5 cm, the attached ovary measured 8.×1.8×0.5 cm, and the salpinx measured 16 cm in length and 0.5 cm in diameter. The left uterus measured 4.7×3.7×2.0 cm, the attached ovary measured 7.0×2.3×1.5 cm, and the salpinx measured 7.2 cm in length and 0.5 cm in diameter. The serosal surface of the uterus, both ovaries and the mesosalpinx had multiple fungating nodules; the largest of which was 1.3 cm in diameter. Both uteri had separate uterine cavities and blind ends. The endometrial cavities of the right and left uteri measured 1.5 and 1.1 cm, respectively. The poorly demarcated yellow-gray tumor mass, measuring 8.0×7.0×5.0 cm, invaded the uterine and rectal walls. The lump of omental tissue measured 38.0× 5.4×2.0 cm and had multiple granular mass-like lesions.

**Figure 2.**
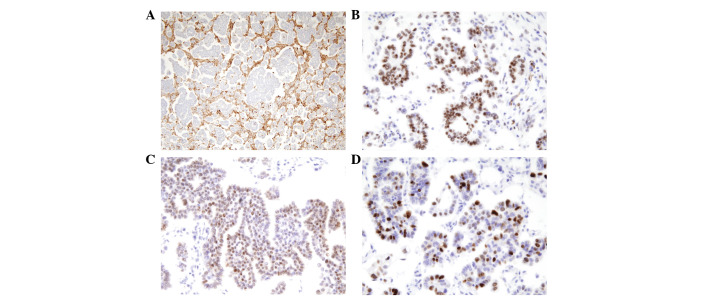
Immunohistochemical staining results were (A) negative for calretinin, but positive for (B) ER, (C) WT-1 and (D) p53.

**Figure 3.**
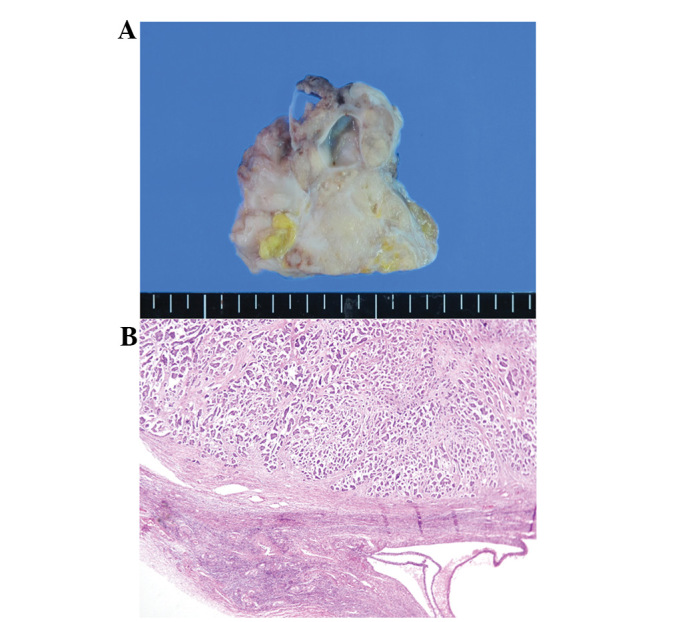
(A) Pelvic tumor section of well-defined ovarian tissue containing cystic follicls shows (B) multiple deposits of serous papillary carcinoma on the surface and superficial cortex (Magnification, ,×40).

Histologically, the main tumor in the peritoneum was a serous papillary carcinoma, characterized by the thin or complex papillary growth of cuboidal-to-columnar serous-type cells. Numerous psammoma bodies were noted (Figs. 1B and C). The tumor cells stained negative for mesothelial cell markers (D2-40 and calretinin), but positive for the Wilms' tumor-1 (WT-1) antibody, the estrogen receptor (ER) and the progesterone receptor (PR) in the immunohistochemical stains. Ki-67 labeling was detected in 60–70% of tumor cells, while p53 was expressed in numerous tumor cells (Fig. 2A–D). The well-defined ovarian tissue containing cystic follicles and corpora albicantia demonstrated tumor involvement in multiple sections of the pelvic mass (Fig. 3A). Both ovaries exhibited normal histological features, but had multiple deposits of serous papillary carcinoma on the surface and superficial cortex (Fig. 3B). The mesosalpinx also contained tumor deposits.

Both uteri had the proliferative phase of the endometrium, and adenomyosis was present in the right-side of the uterus.

## Discussion

MRKH syndrome, first described by Mayer and further studied by Rokitansky, Küster and Hauser, is one spectrum of Müllerian anomalies originally characterized by the congenital absence of a uterus and vagina in genotypic and phenotypic females with a normal endocrine status (4,5). MRKH syndrome affects 1 in 4,000 live female births, and is the second most common cause of primary amenorrhea (6).

In isolated MRKH syndrome (type I), the Fallopian tubes and ovaries are usually present, as well as variable degrees of uterine or vaginal aplasia. Type II MRKH syndrome includes other non-Müllerian anomalies, such as urinary tract and cervicothoracic disorders, and hearing defects (7). The evaluation of renal defects is mandatory, as anomalies such as the hypoplastic and non-functioning left kidney in the study by Capero and Gallego are common (6). The patient in the present case also showed a scoliotic change in the thoracic spine; however, a supernumerary ovary is an extremely unusual accompanying anomaly with type II MRKH syndrome.

During surgery, the pelvic mass was suggested to be a primary peritoneal carcinoma, as the ovaries had been defined to be morphologically normal. The primary peritoneal serous carcinoma was histologically indistinguishable from a primary ovarian serous tumor. We propose that malignant mesothelioma arising from the peritoneum ought to be distinguished from primary peritoneal Müllerian neoplasms. Immunohistochemical staining for mesothelial markers (D2-40 and calretinin) excluded the mesothelial origin. The expression of ER and PR, as well as WT-1, favored a serous tumor of Müllerian origin.

The distinction between a supernumerary and an accessory ovary is not always clear. In an extensive review of the literature in 1959, Wharton described the supernumerary ovary (8). The term ‘supernumerary ovary’ is used to include rare cases of ectopy in which the third ovary is entirely separate from the ovary which is situated normally. The term ‘accessory ovary’ includes cases in which excess ovarian tissue is situated near the normally placed ovary, and may have a connection with it, appearing to have been developed from it (9).

There are two proposed mechanisms for the formation of a supernumerary ovary (10). Pearl and Plotz proposed the arrested gonocyte migration theory. Gonocytes may be arrested as they pass retroperitoneally through the dorsal mesentery (9). Alternatively, the transplantation theory of the germinal ridge following incorporation of the gonocyte was proposed by Printz *et al*(11). Supernumerary ovaries may be situated in the pelvis, the retroperitoneum, the para-aortic area or the colonic mesentery. Supernumerary ovaries may be multiple and functional, associated with ovarian neoplasms or located with other congenital malformations of the genitourinary system (12). The mass may present as either painful or asymptomatic, and is often found incidentally during surgery or autopsy.

From these mechanisms, we suggest that the supernumerary ovary tends to be situated in the posterior peritoneum, where it is hard to detect the normal-sized ovary without expectation, compared with the anterior peritoneum. This explains why the third ovary was not identified in the first diagnostic scope operation in the present case.

In summary, we report the first case of cancer of the super-numerary ovary in a patient with MRKH syndrome. Although both ovaries were confirmed to be normal in the present patient with MRKH syndrome, an ovarian neoplasm should be considered in pelvic mass diagnosis.
